# First Study of the Prevalence and Characterization of Brachial Plexus Injuries in Guatemala

**DOI:** 10.1055/s-0041-1731746

**Published:** 2021-07-27

**Authors:** Carmen Joanna González Lemus, Fernando Xavier Romero Prieto

**Affiliations:** 1 Department of Medical Science, Rafael Landivar University of Guatemala, Guatemala City, Guatemala; 2 Brachial Plexus and Peripheral Nerve Unit, Department of Orthopedics and Traumatology, Roosevelt Hospital, Guatemala City, Guatemala

**Keywords:** brachial plexus, injury, prevalence, characterization, upper extremity

## Abstract

**Objective** This study aimed to estimate the prevalence of brachial plexus injuries and to characterize clinically and epidemiologically patients with brachial plexus injury.

**Materials and Methods** In this cross-sectional descriptive study, 2,923 medical records of patients aged 1 to 64 years who presented at outpatient peripheral nerve unit of the Orthopedic Surgery Department of Hospital Roosevelt, Guatemala, from January 2017 to December 2017, were prospectively analyzed to identify the prevalence and factors associated with brachial plexus injuries.

**Results** The prevalence rate of brachial plexus injuries in patients was 5.74%. This injury is more common in men (90.5%) aged 24 to 64 years. Brachial plexus injuries occurred secondary to motorcycle accident in 72% of the cases, with the majority affecting the dominant upper extremity. In addition, 64.28% of the patients took 1 to 6 months to seek consultation, whereas only 16.07% requested medical assistance <1 month from the onset of symptoms, and this result was associated with early diagnosis and adequate recovery during follow-up. Furthermore, 66.67% presented upper brachial plexus injury with no associated fractures or vascular injury, manifesting distress while performing daily activities that required hand, arm, and elbow movements.

**Conclusion** The risk of suffering BPIs in Guatemala increases in economically active male patients that use motorcycles as main mode of transportation. Patients should consult immediately after injury onset to optimize management results. For this reason, hospitals must develop specialized clinical guidelines to speed up the identification and treatment of BPI injuries.

## Introduction

The brachial plexus is a complex anatomical structure that originates in the neck region and branches from the spinal cord to form other nerves that control movement and sensation of the shoulder, arm, forearm, and hand; therefore, a traumatic injury occurs when one or more of these nerves is pulled, compressed, or torn. Among the main causes are motorcycle and vehicle accidents, gunshot wounds, work-related accidents and, to a lesser extent, sports. 1 2 3

These injuries result in the loss of mobility, sensitivity, and chronic pain when they are not treated early. Untreated injuries influence the patient's quality of life due to limited movement of the upper limb, which eventually leads to termination from work, generating stress, anxiety, and depression. Patients also tend to isolate themselves or hide their arm because they feel ashamed of its appearance; their self-esteem is thus affected. 2 4

According to a study conducted in Colombia in 2013, 90% of persons who encountered motorcycle accidents sustained brachial plexus injury (BPI), and accidents were mostly related to alcohol intake. In Uruguay, a country with a high percentage of motorcycle use, in 2016, 90% of patients with blunt trauma had BPI, and of these patients, 80% were men aged 25 to 35 years, and the same male percentage has been found in studies performed in Germany, Egypt, and Brazil. 4 5 6

In Guatemala, no statistical data are available on these types of injuries; however, according to the office of the peripheral nerve unit of the Department of Traumatology and Orthopedics in January 2017, 29 patients, mostly men, were recorded to have BPI, and the main causes were motorcycle accidents, bullet wounds, and stab wounds.

Traumatic BPI is repairable when it is detected and surgically treated in the first 6 months of injury onset. 6 7 In Guatemala, in the area of medical public health, Hospital Roosevelt is the only medical center in the public network that has a unit, which was created 4 years ago, with medical and surgical equipment to treat BPIs.

This study aimed to investigate the prevalence and to characterize clinically and epidemiologically patients with BPI in Hospital Roosevelt, Guatemala.

## Materials and Methods

The study was conducted in the outpatient peripheral nerve unit and brachial plexus unit of the Orthopedics and Traumatology Department, Hospital Roosevelt, Guatemala, from January 1, 2017 to December 31, 2017, and 168 records with diagnosis of BPI were selected.

### Statistical Analysis

A template was prepared in Microsoft Excel 2016, and data obtained from medical records were entered into spreadsheets. Data were organized in tables and graphs and presented as frequencies, percentages, with confidence interval of 95%, which was calculated to analyze information based on the objectives.

## Results

### General Prevalence of Brachial Plexus Injury at Hospital Roosevelt, Guatemala

The prevalence of BPIs was 5.74%, which was higher in February and April, and the monthly prevalence was higher than the general prevalence of 8.93 and 8.47%, respectively. In April, only one patient aged <1 year presented to the peripheral nerve unit throughout the year ( Table 1 ).

**Table 1:** Prevalence of brachial plexus injury in adult patients who presented to the outpatient peripheral nerve and brachial plexus unit of the Orthopedics and Traumatology Department of Hospital Roosevelt, Guatemala in 2017

Months	Total patients with other injuries	Total patients with BP lesion	Total patients who sought consultation	Prevalence (%)
January	233	20	253	7.90
February	204	20	224	8.93
March	257	20	277	7.22
April	227	21	248	8.47
May	264	20	284	7.04
June	273	11	284	3.87
July	271	11	282	3.90
August	195	7	202	3.46
September	154	12	166	7.23
October	276	10	286	3.50
November	266	7	272	2.57
December	135	9	145	6.21
Total	2,755	168	2,923	5.74

Abbreviation: BP, brachial plexus.

Note: Own elaboration based on data collection form 1 ( *n* = 2,923).

Among patients who sought treatment, 141 adults (aged 20–64 years) and 17 adolescents visited the unit for BPI treatment ( Table 2 ).

**Table 2:** Age (years) of patients who consulted in the outpatient peripheral nerve and brachial plexus unit of the Orthopedics and Traumatology Department of Hospital Roosevelt, Guatemala in 2017

Age range (y)	Patients	CI (−)	CI (+)
0–1	1	−0.57	1.76
1–11	0	0	0
12–19	24	5.56	14.68
20–64	141	78.37	89.48
>64	2	−0.45	2.83

Abbreviation: CI, confidence interval.

Note: Own elaboration based on data collection form 1 ( *n* = 168).

With regard to sex, 152 (90.5%) men and 16 (9.5%) women presented this condition ( Table 3 ).

**Table 3:** Sex distribution of adult patients who consulted the outpatient peripheral nerve and brachial plexus unit of the Orthopedics and Traumatology Department, Hospital Roosevelt, Guatemala in 2017

Sex			CI (−)	CI (+)
Male	152	90.5%	86	95
Female	16	9.5%	5	14

Abbreviation: CI, confidence interval.

Note: Own elaboration based on data collection form 1 ( *n* = 168).

As regards the interval from date of injury to date of consultation, 28 patients presented to the unit for BPI within 1 month from injury, 52 between 1 and 3 months, 56 took 3 to 6 months, 25 waited 6 months to 1 year, and 8 waited >1 year ( Table 4 ).

**Table 4:** Delay time from date of injury to patient consultation at the outpatient peripheral nerve and brachial plexus unit of the Orthopedics and Traumatology Department of Hospital Roosevelt, Guatemala in 2017

Time until consultation	Number of patients		CI (−)	CI (+)
1 mo	27	16.07%	1	22
1–3 mo	52	30.95%	2	38
3–6 mo	56	33.33%	3	40
6 mo to 1 y	25	14.88%	1	20
>1 y	8	4.76%	0	8

Abbreviation: CI, confidence interval.

Note: Own elaboration based on data collection form 1 ( *n* = 168).

The medical records of patients who presented to the outpatient peripheral nerve and brachial plexus unit of the Orthopedics and Traumatology Department of Hospital Roosevelt, Guatemala were reviewed, and a total of 2,923 patients presented for consultation from January 2017 to December 2017, of which 168 patients sought treatment for BPIs, including one child and 167 adults.

The prevalence is higher in February and April. The infant prevalence of BPIs was 0.40%; throughout the year (April), only one child presented for BPI.

As regards the mechanism of injury, 64.3% of the patients sustained BPI following a motorcycle accident, and 18.5% following a car accident. Most of the BPIs are caused by street-related accidents ( Table 5 ).

**Table 5:** Mechanism of injury in adult patients who consulted to the outpatient peripheral nerve and brachial plexus units of the Orthopedics and Traumatology Department, Hospital Roosevelt, Guatemala in 2017

Mechanism of injury		
Motorcycle accident	108	64.3%
Car accident	31	18.5%
Falls	13	7.7%
Assaults	10	6%
Bicycle accident	3	1.8%
Run-over	2	1.2%
Obstetrics	1	0.6%

Note: Own elaboration based in data collection ( *n* = 168).

Based on data collected during physical examination of all patients diagnosed with BPI, 66.67% had an upper lesion and 22.6% had total lesion ( Table 6 ).

**Table 6:** Type of brachial plexopathy in patients who consulted to the outpatient clinic of the Traumatology and Orthopedics Department in 2017

Level of brachial plexus injury		
Upper injury	112	66.67%
Lower injury	18	10.71%
Total injury	38	22.61%

Note: Own elaboration based in data collection ( *n* = 168).

## Analysis and Discussion

In this study, 83.93% of the patients with BPI belonged to the 20 to 64 age group (95% confidence interval [CI]: 78–89), and 10.1% belonged to the 12 to 19 age group (95% CI: 6–15). This finding suggest that adults are at a higher risk for BPI, since they are engaged in higher risk jobs ( *p* = 1.2635 e-08). In an epidemiological study performed in Brazil, Faglioni et al reported a mean age of 28.38 years. 8

Up to 95% of the cases were recorded in men and 10% in women and this result is consistent with the literature, international studies have described that BPI occurs more often in men. 8 9 However, in the analysis using Chi-square test, women aged <18 years are more susceptible to BPI ( *p* = 0.00024164); however, men have the highest occupational risk ( *p* = 1.4395 e-06), that is, men more often sustain injury due to nonwork activities such as driving. For this reason, a strong association was found between men and use of vehicle to get around ( *p* = 0.02698), with vehicle accidents as the main cause of most BPIs.

In this study, 54% (95% CI: 47–62) of the patients have a partner, and 46% (95% CI: 38–53) do not have a partner. This result indicates that the majority of the patients have a family, and most of these patients were men who provided financial support to their family. The occurrence of BPI in this population affects both socioeconomic aspect and family life, since in Guatemala the majority of the population work on their own initiative, that is, they were only paid by the number of days worked. Therefore, early and timely detection of BPI is important to avoid sequelae that cause permanent disability.

The occupations of patients who consulted for PBIs were also investigated to determine whether an occupational risk is associated with BPI. In this study, 20.8% ( *n* = 35) were messengers, 16.1% ( *n* = 27) were students, 10.1% ( *n* = 17) were food delivery workers, 8.9% ( *n* = 15) were pilots, 8.3% ( *n* = 14) were maintenance workers, and 8.3% ( *n* = 14) were construction workers, the highest percentage being the messengers. Notwithstanding the occupational risk, these lesions are more related to the means of transportation, by which most used motorcycles (64.3%). This result coincides with those of studies performed in Brazil, Uruguay, Egypt, and Colombia, where the majority of the patients who presented with this injury used motorcycles as a means of transportation. 5 8 9

As regards the interval from injury onset to consultation date, 80.35% of the patients sought treatment from 1 to 6 months. Therefore, most of the patients sought medical assistance before the 6-month time limit, which was reported to be associated with favorable surgical outcomes. 10

In this cross-sectional study, 54% of the patients with BPI did not have associated fractures, while 46% presented fractures. This finding is related to the long waiting time until consultation because there were initially no visible injuries or activity limitations. Those with fractures were treated for related bone injuries; however, they were not evaluated by BPIs for this reason. This finding is different from the results of Li et al in Guangxi, China, where 76.27% of the cases had accompanying fractures. 11

In this study, 81% of the patients did not suffer from any comorbidity detected upon consultation. The comorbidities recorded among the 19% were arterial hypertension and type 2 diabetes mellitus. These comorbidities were reported in patients who worked as messengers or construction workers and were related infallibly with poor eating habits.

In this study, 66.67% of the patients had an upper extremity BPI, which causes difficulty in performing activities of daily living, since it affects the mobility of the shoulder and elbow. Moreover, 22.61% presented a total BPI, in which patients not only lost upper limb mobility, but also suffered from significant pain that interferes with daily life and is the main reason for consultation. In addition, 10.71% of the patients presented with a lower BPI that does not allow hand mobility.

In patients with BPI, 90% had dominant limb injury, with 60% affecting the right arm. Thus, BPI generally occurs in the dominant limb in cases with direct contusions.

An electromyogram was performed on all patients as a complementary diagnostic study, and no other examinations were performed due to the high costs of more complex tests such as magnetic resonance imaging.

In this study, 20% of the patients who consulted the outpatient facility reported a history of previous surgeries for associated injuries such as fractures and/or vascular lesions occurred at the time of the initial traumatism.

Moreover, 48.21% had skin lesions and soft tissue injuries, with pain as the main complaint after the first month of injury. Muscle pain referred in these patients is associated with considerable work limitation, which explains the need for consultation.

Most patients took 1 to 6 months to receive an adequate diagnosis and subsequent treatment. Notably, most of the patients were referred from different hospitals in the national public health system or private hospitals, and all had already consulted more than one doctor for the same injury.

## Conclusions

- The general prevalence of BPIs in the Hospital Roosevelt of Guatemala is up to 5.74%.
- Injuries are mostly caused by motorcycle and vehicle accidents.
- The populations at risk for BPIs are economically active male adults who travel by motorcycle, with less regard for the risk level of their occupation.
- Most common injuries were upper BPIs, these injuries frequently developed in the dominant extremities.

## Recommendations

- Further studies should take into account other hospitals and other regions of the country where a larger database can be generated and results can be compared.
- Subsequent study to assess the prevalence of obstetrical brachial plexus paralysis in Guatemala.
- Further study should investigate the efficacy of treatments offered to patients after they have been evaluated and diagnosed in the Department of Traumatology and Orthopedics of Hospital Roosevelt.
- Study results should be disseminated widely to medical staff, residents of traumatology and orthopedics, emergency service, and other relevant departments of Hospital Roosevelt so that injuries are diagnosed early and thus sequelae are reduced.
